# Optimizing Outcomes in Vertebral Fractures: The Impact of Intercostal Nerve Block on Costal Pain

**DOI:** 10.1155/joos/5595038

**Published:** 2025-12-18

**Authors:** Ziyao Ma, Xuelian Peng, Shuang Xu, Qing Wang, Shuai Zhang

**Affiliations:** ^1^ Southwest Medical University, Luzhou, 646000, Sichuan, China, swmu.edu.cn; ^2^ Department of Ultrasound, The Affiliated Hospital of Southwest Medical University, Luzhou, 646000, Sichuan, China, ahswmu.cn; ^3^ Department of Orthopedics, The Affiliated Hospital of Southwest Medical University, Luzhou, 646000, Sichuan, China, ahswmu.cn

**Keywords:** costal pain, disease duration, intercostal nerve block, osteoporotic vertebral compression fracture, percutaneous vertebroplasty

## Abstract

**Objective:**

To assess the effectiveness of intercostal nerve block (INB) for treating osteoporotic vertebral compression fractures (OVCFs) with associated costal pain.

**Methods:**

We reviewed clinical data from patients with thoracic OVCF and costal pain admitted to our hospital between January 2021 and January 2024. Patients were divided into an observation group, receiving percutaneous vertebroplasty (PVP) and INB, and a control group, receiving PVP alone. Baseline data, intraoperative parameters, deformity improvement (anterior vertebral body height [AVH] and local Cobb angle), and clinical symptom improvement (visual analog scale [VAS] scores for back and costal pain) were compared between the groups. Risk factors for residual costal pain within the control group were analyzed using multivariable logistic regression, and receiver operating characteristic (ROC) curves were constructed to determine threshold values for the identified risk factors.

**Results:**

The study included 305 patients, 150 in the observation group and 155 in the control group. The groups were statistically comparable in baseline data. Compared with the control group, the observation group had a longer operative time (40.7 ± 5.5 vs. 32.4 ± 3.8 min, *p* < 0.001) and required more intraoperative C‐arm fluoroscopies (30.4 ± 6.3 vs. 21.5 ± 3.9, *p* = 0.034). Intraoperative bleeding was similar between groups. Both groups showed similarly significant improvements in AVH, local Cobb angle, and thoracic back pain VAS scores one day postoperatively and at the final follow‐up. The respective costal pain VAS scores in the control and observation groups were as follows: preoperatively, 7 (6, 8) and 7 (6, 7); one day postoperatively, 4 (2, 5) and 2 (1, 2); and at the final follow‐up, 1 (1, 2) and 1 (0, 2). Univariate analysis within the control group identified disease duration, fractured vertebral body width, reduced intervertebral foramen area, and cortical breakdown of the vertebral body’s posterior wall as risk factors for residual costal pain. Multivariable analysis confirmed disease duration as an independent risk factor for residual costal pain, with an area under the curve of 0.863. The threshold for self‐resolution of costal pain was established at 15.5 days, with a sensitivity of 93.9% and a specificity of 70.0%. Costal pain relief was strongly correlated with disease duration (*r* = 0.518, *p* < 0.001).

**Conclusions:**

OVCF‐related costal pain can be effectively alleviated by PVP combined with INB; however, INB lengthens operative time and increases radiation exposure. PVP alone can relieve costal pain in patients with a disease duration of ≤ 15.5 days; otherwise, concomitant INB is recommended.

## 1. Background

With the aging of society, osteoporosis has gradually become one of the most important chronic diseases threatening the health of older adults. According to the World Health Organization, 6.3% of men and 21.2% of women over the age of 50 have osteoporosis globally [1]. In China, the prevalence of osteoporosis among people over 65 years of age is 32.0% (10.7% in men and 51.6% in women) [2]. Among these women, 40.0% are expected to experience a fragility fracture during their lifetime, placing a substantial burden on society and families [3, 4].

Osteoporotic vertebral compression fractures (OVCFs) represent the most prevalent complication associated with osteoporosis [5, 6]. The primary clinical manifestation of OVCFs is mechanical low back pain, typically localized to the level of the fractured vertebra near the midline, hence termed “midline pain” [7–10]. Conversely, Gibson et al. [10] described pain in the quaternary rib, hip, and groin regions as “non‐midline pain” associated with OVCFs. Notably, the thoracic spine is the most frequent site of OVCFs. Approximately one‐third of patients with thoracic OVCFs experience unilateral or bilateral costal pain, with some presenting this as their predominant clinical symptom [11–13]. Thoracic OVCFs are often characterized by vertebral fracture–associated unilateral or bilateral costal pain. It is important to exclude other causes such as rib fractures, intercostal neuritis, shingles, and other pathologies when diagnosing the source of this pain. This pain type is often exacerbated by deep breathing, sneezing, or posture changes [10, 13, 14].

Previous research has identified several etiological factors contributing to thoracic OVCF‐related costal pain [11, 13–15]. These primarily include stimulation of the sympathetic ganglia surrounding the fractured vertebra, intervertebral foraminal stenosis, loss of articular stability of the articular processes, increased fractured vertebral body width, and paraspinal muscle atrophy [10, 11, 13–15]. The vertebral fracture itself constitutes the central pathological alteration underlying these factors. Consequently, Gibson and Lin [10, 11] advocated reconstruction of spinal stability through percutaneous vertebroplasty (PVP) as an indirect means of alleviating costal pain. However, approximately 20% of patients continue to experience residual costal pain postoperatively [10, 16]. Some researchers have attributed this persistent pain to inadequate restoration of the intervertebral foraminal height or challenges in eliminating soft tissue compression around the intercostal nerve [10, 13, 16].

Intercostal nerve blocks (INBs) are recommended for various chronic and acute pain conditions, including rib fractures, shingles, postthoracotomy pain syndrome, and intercostal neuralgia [17–19]. However, to our knowledge, there are no published reports describing the use of INB to treat OVCF‐related costal pain. This study aimed to evaluate the efficacy of combining INB with PVP in managing OVCF with associated costal pain. Additionally, it sought to identify risk factors associated with residual costal pain in patients with OVCF and concurrent costal pain treated with PVP alone.

## 2. Methods

### 2.1. Patient Selection

The ethics committee of our hospital approved this retrospective study (KY2025286) and waived the requirement for patient‐informed consent due to its retrospective nature. The study complied with the Declaration of Helsinki and the Institutional Review Board (IRB) guidelines. Clinical data from patients with thoracic OVCF accompanied by costal pain who were admitted to our hospital from January 2021 to January 2024 were reviewed and divided into two groups: an observation group (PVP combined with INB) and a control group (PVP alone). The inclusion criteria were as follows: (1) age ≥ 60 years or bone mineral density (BMD) *T*‐score ≤ −2.5 as measured by dual‐energy X‐ray absorptiometry; (2) a known history of hypochondriac and/or back pain, with or without limited mobility; (3) an acute, single thoracic OVCF confirmed by magnetic resonance imaging; (4) costal pain at the fractured vertebral level; and (5) follow‐up for at least 12 months after surgery, with the most recent X‐ray examination performed at least 12 months postoperatively. The exclusion criteria were as follows: (1) Fractures caused by pathological conditions such as primary or secondary tumors or vertebral infections; (2) vertebral fractures compressing the spinal cord; (3) vertebral body‐occupying lesions; (4) multisegmental fractures; and (5) concomitant rib fractures.

### 2.2. Surgical Methods

All PVP procedures were performed by two spinal surgeons (W.S. and X.S.) who had received standard training in PVP surgery and had more than 10 years of procedural experience. A unilateral puncture under local anesthesia was used for all patients. The fractured vertebra was localized using C‐arm fluoroscopy, and the entry point was marked at the midpoint of the transverse process, 3–5 mm lateral to the outer edge of the pedicle projection. A successful puncture was confirmed when the needle extended to the inner edge of the pedicle on an anteroposterior (AP) view and the needle tip was positioned at the posterior margin of the vertebral body on the lateral view. A guidewire was implanted if the needle extended 5 mm anterior to the posterior margin of the vertebral body. Guidewires positioned in the anterior one‐third of the vertebral body on a lateral view but reaching or exceeding the midline of the spinous process on the AP view were replaced with a bone‐cement injection catheter. A 3‐mm drill was inserted through the cannula, confirming its position at the anterior one‐third of the vertebral body under lateral fluoroscopy. After drill removal, bone cement was incrementally injected according to time‐ and temperature‐gradient principles. C‐arm lateral fluoroscopy was used to monitor the injection process. The plunger was promptly detached from the cement, and the working cannula was rotated after cement delivery. Once the cement solidified, the cannula was withdrawn. If unilateral puncture failed to achieve adequate cement dispersion, a contralateral pedicle puncture was performed to supplement the initial injection.

Patients in the observation group underwent INB immediately after the PVP procedure, guided by the C‐arm to the intercostal space. The puncture site was located at the costal angle on the inferior margin of the rib corresponding to the fractured vertebra, with care taken to avoid intercostal vessels. The puncture needle was advanced until it contacted the rib and then maneuvered downward along the rib surface. Once the needle tip passed the lower rib edge, it was further inserted by 2‐3 mm. After confirming the absence of blood or gas upon aspiration, the patient was instructed to hold their breath while a 3.0‐mL premixed ropivacaine hydrochloride and triamcinolone acetonide acetate was injected. The mixture was prepared by combining one 10‐mL vial of 100 mg/mL ropivacaine hydrochloride injection and one 5‐mL vial of 50 mg/mL triamcinolone acetonide acetate injection and then diluting the mixture with normal saline to a total volume of 21 mL. This yielded final concentrations of 0.48% for ropivacaine hydrochloride and 0.24% for triamcinolone acetonide acetate [20]. Patients in the control group who experienced residual costal pain postoperatively underwent supplementary INB or received oral anti‐inflammatory and analgesic medications during follow‐up, depending on their reported pain levels and treatment preferences.

All surgical procedures were performed by S.X., who underwent professional training and has over a decade of experience in spinal surgery. All patients used back braces for 1‐2 months after the procedure and were treated with antiosteoporotic medications.

### 2.3. Evaluation of Clinical Parameters

Clinical parameters included baseline characteristics such as sex, age, disease duration, and BMD. Intraoperative parameters included surgery duration, intraoperative blood loss, frequency of C‐arm fluoroscopy use, and incidence of bone‐cement leakage. The clinical efficacy of the two surgical procedures was evaluated by changes in visual analog scale (VAS) scores determined preoperatively, one day postoperatively, and at the final follow‐up. Two independent third‐party evaluators conducted baseline and follow‐up VAS assessments. The mean of the two evaluators’ scores was used for intergroup comparisons.

### 2.4. Evaluation of Radiological Parameters

Anterior vertebral body height (AVH) and local Cobb angle (CA) were determined as described by Lee et al. and Kuklo et al., respectively, and recorded at the same three time points.

The injured vertebral width ratio was assessed following Xin et al. [14]. Once the sagittal center of the damaged vertebral body was identified, the lower edge of the injured pedicle served as a reference point for measuring the maximum width of the injured vertebral body on the coronal plane. If the fracture involved only the upper or lower portion of the vertebral body, the two surgeons jointly selected the more appropriate coronal plane to record the maximum width. The reduction in foraminal area was measured following Chen et al. [13]. In the injured vertebral body, the upper and lower foramina were denoted as A and C, respectively, and the foramen area at the injured vertebral level was denoted as B. The preinjury foramen area was calculated as (A + C)/2 and the reduction area as (A + C)/2 − B [13]. The presence of a fracture in the posterior wall of the vertebral body was determined using sagittal and coronal CT 3D reconstruction images.

### 2.5. Analysis of Risk Factors for Residual Costal Pain

Based on existing literature on risk factors associated with OVCF‐related costal pain, we compared patients within the control group who experienced remission of costal pain with those who did not, to identify differences in these factors. Significant factors identified through univariate logistic regression analysis were included in the multivariable logistic regression analysis, and receiver operating characteristic (ROC) curves were constructed. These were used to calculate threshold values for risk factors of residual costal pain following PVP and to examine correlations between residual costal pain and independent risk factors.

### 2.6. Analysis of Complications

The incidence of complications, including cement leakage, surgical site infection, adjacent vertebral fracture, and hemopneumothorax, was compared between the groups.

### 2.7. Statistical Analysis

All data were statistically analyzed using IBM SPSS Statistics for Macintosh, Version 29.0 (IBM Corp., Armonk, NY, USA). The Shapiro–Wilk test for normality assessed the distribution of continuous variables. Normally distributed variables are expressed as means ± standard deviations, nonnormally distributed variables are expressed as medians (interquartile range), and categorical variables are expressed as counts and percentages. The independent‐samples *t*‐test was used to compare age, follow‐up time, BMD, disease duration, operative time, intraoperative bleeding, AVH, local CA, vertebral body width, and intervertebral foraminal reduction area between groups. The chi‐squared test compared sex composition, fracture site, and the presence or absence of cortical disruption in the posterior wall of the vertebral body. The Wilcoxon signed‐rank test was used for two‐way comparisons of VAS scores, with a corrected two‐sided significance level of *α* = 0.05. Univariate logistic regression analysis was used to identify associated risk factors. Significant variables from the univariate analysis were included in the multivariable logistic regression analysis model. Independent risk factors were analyzed to construct ROC curves, calculate their threshold values leading to possible residual costal pain after PVP, and assess correlations between residual costal pain and identified independent risk factors. Statistical significance was set at *p* < 0.05.

## 3. Results

### 3.1. Baseline Information

The observation (*n* = 150) and control (*n* = 155) groups were statistically comparable in their baseline data, including age, sex, BMD, follow‐up duration, and fracture site (*p* > 0.05; Table 1).

**Table 1:** Baseline data.

Variable	CG (*n* = 155)	OG (*n* = 150)	*p* value
Age (Y)	72.1 ± 5.5	73.3 ± 5.9	0.989

Sex (M)	47 (30.3)	43 (28.7)	0.751
(F)	108 (69.7)	107 (71.3)	

Average follow‐up time (months)	16.3 ± 10.2	17.4 ± 9.0	0.183

BMD (g/cm^2^)	−3.4 ± 1.5	−3.3 ± 2.1	0.135

Duration (d)	29.3 ± 6.8	28.2 ± 4.3	0.194

Fractured vertebrae	155 (100)	150 (100)	0.941
T6	26 (16.8)	27 (18.0)	
T7	27 (17.4)	29 (19.3)	
T8	17 (11.0)	18 (12.0)	
T9	35 (22.6)	31 (20.7)	
T10	22 (14.2)	16 (10.7)	
T11	28 (18.1)	29 (19.3)	

*Note:* M, male, F, female.

Abbreviations: BMD, bone mineral density; CG, control group; OG, observation group.

### 3.2. Intraoperative Parameters

The surgical duration (40.7 ± 5.5 vs. 32.4 ± 3.8 min; *p* < 0.001) and the number of intraoperative C‐arm fluoroscopy uses (30.4 ± 6.3 vs. 21.5 ± 3.9; *p* = 0.034) were significantly greater in the observation group than in the control group. Intraoperative blood loss and bone‐cement injection volume were statistically comparable in the two groups (Table 2).

**Table 2:** Intraoperative parameters.

Group	Operative time (min)	Blood loss (mL)	Fluoroscopy	Bone‐cement volume (mL)
CG	32.4 ± 3.8	16.7 ± 9.6	21.5 ± 3.9	4.0 ± 5.1
OG	40.7 ± 5.5	17.9 ± 8.3	30.4 ± 6.3	4.2 ± 4.8
*χ* ^2^/*t*/*z*	−22.643	4.454	−0.909	1.023
*p* value	< 0.001	0.486	0.034	0.243

### 3.3. Clinical Findings

The groups had comparable VAS scores for both back pain (*p* = 0.832) and costal pain (*p* = 0.854). Both groups showed similarly significant improvements in thoracic back pain VAS scores one day postoperatively and at the final follow‐up. The respective costal pain VAS scores in the control and observation groups were as follows: preoperative, 7 (6, 8) and 7 (6, 7); one day postoperatively, 4 (2, 5) and 2 (1, 2); and at the final follow‐up, 1 (1, 2) and 1 (0, 2). In the control group, preoperative costal pain decreased significantly (by ≥ 2 points on the VAS) in 101 patients. The remaining 54 patients had residual costal pain, accounting for the significantly higher costal pain scores in the control group compared with the observation group one day postoperatively. During follow‐up, 38 patients selected INB for intolerable costal pain, while 16 opted for conservative treatments such as oral nonsteroidal anti‐inflammatory and analgesic medications. At the final follow‐up, the VAS scores of patients with residual costal pain in the control group were significantly lower than their preoperative values and statistically comparable to those in the observation group (Table 3).

**Table 3:** Clinical parameters.

Group	VAS of costal pain			VAS of back pain		
	Preoperative	1 d postoperative	Final follow‐up	Preoperative	1 d postoperative	Final follow‐up
CG	7 (6.8)	4 (2.5)	1 (1.2)	7 (6.7)	3 (2.3)	1 (1.2)
OG	7 (6.7)	2 (1.2)	1 (0.2)	7 (6.8)	2 (2.4)	1 (1.2)
*z*	−1.468	−10.606	−0.144	−1.532	−1.386	−1.336
*p* value	0.142	< 0.001	0.885	0.236	0.198	0.685

Abbreviation: VAS, visual analog scale.

### 3.4. Radiological Findings

Both groups had statistically comparable preoperative, one‐day postoperative, and final follow‐up local CA and AVH values of the vertebral body and demonstrated similarly significant postoperative improvements in both measures (Table 4).

**Table 4:** Kyphosis improvement parameters.

Group	Cobb (°)			AVH (mm)		
	Preoperative	1 d postoperative	Final follow‐up	Preoperative	1 d postoperative	Final follow‐up
CG	11.5 ± 2.9	7.4 ± 5.2	7.6 ± 4.1	18.4 ± 2.9	22.7 ± 3.1	22.0 ± 4.1
OG	11.9 ± 3.5	7.7 ± 4.3	8.0 ± 3.8	18.1 ± 4.1	21.9 ± 4.0	21.5 ± 2.8
*t*	1.012	−0.447	−0.550	−0.726	−1.830	−0.367
*P*	0.312	0.655	0.583	0.468	0.068	0.714

Abbreviation: AVH, anterior vertebrae height.

### 3.5. Analysis of Risk Factors for Residual Costal Pain

**Figure 1:**
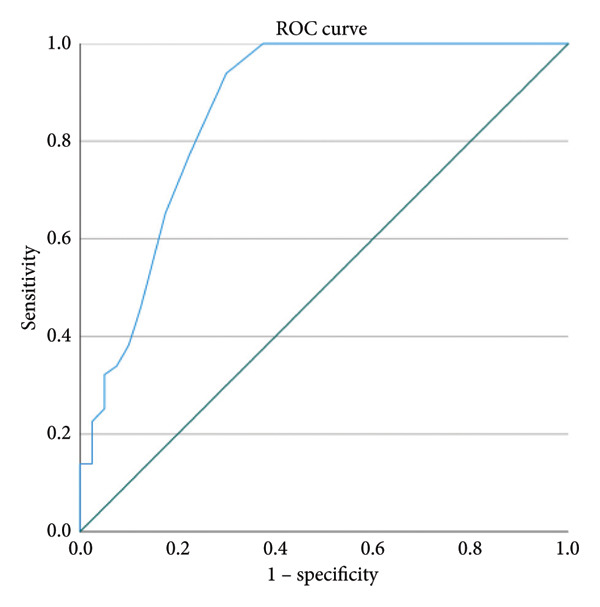
The area under the curve (AUC) for disease duration.

Based on previous literature on risk factors associated with OVCF‐related costal pain, we conducted a comparative assessment between patients with (*n* = 101) and without (*n* = 54) postoperative relief from costal pain. Significant differences were found in disease duration, fractured vertebral body width, intervertebral foraminal reduction area, and posterior vertebral body wall fracture between these two groups (Table 5). Multivariable logistic regression analysis identified disease duration as an independent risk factor for residual costal pain in the control group (Table 6). The area under the curve (AUC) for disease duration was 0.863. A disease duration threshold of 15.5 days for postoperative self‐resolution of costal pain yielded a sensitivity of 93.9% and a specificity of 70.0% (Figure 1). A strong correlation was observed between the degree of self‐resolution of costal pain and disease duration (*r* = 0.518, *p* < 0.001).

**Table 5:** Analysis of risk factors for residual costal pain.

Variable	Residual costal pain (*n* = 54)	Cost pain relief (*n* = 101)	*p* value
Age (Y)	71.7 ± 5.7	72.6 ± 5.2	0.932

Sex (M)	11 (20.4)	23 (22.8)	0.716
(F)	43 (79.6)	78 (77.2)	

BMD (g/cm^2^)	−3.4 ± 2.5	−3.3 ± 3.7	0.185

Duration (d)	31.2 ± 4.8	10.9 ± 4.1	< 0.001

Cobb (°)	12.1 ± 6.1	11.7 ± 5.2	0.344

AVH (mm)	18.6 ± 3.4	18.3 ± 4.9	0.448

Vertebral width (mm)	21.4 ± 4.5	19.2 ± 2.6	< 0.001

Reduced area of intervertebral foramen (mm^2^)	11.1 ± 2.5	9.9 ± 3.4	0.014

Vertebral posterior wall fracture (Yes)	34 (63.0)	31 (44.3)	< 0.001
(No)	20 (37.0)	70 (55.7)	

Fractured vertebrae	54 (100)	101 (100)	0.922
T6	6 (11.1)	14 (13.9)	
T7	7 (13.0)	16 (15.8)	
T8	7 (13.0)	10 (9.9)	
T9	15 (27.8)	20 (19.8)	
T10	8 (14.8)	20 (19.8)	
T11	11 (20.3)	21 (20.8)	

**Table 6:** Multivariate logistic regression analysis of residual costal pain.

Variable	*B*	*P*	OR	5% CI	95% CI
Duration	−1.045	< 0.001	0.352	0.193	0.64
Vertebral width	2.473	0.066	11.853	0.848	16.708
Reduced area of intervertebral foramen	0.072	0.097	1.027	0.995	1.061
Vertebral posterior wall fracture	−2.186	0.060	0.112	0.011	1.100

### 3.6. Complications

The two groups had statistically comparable complication rates. Instances of cement leakage and displacement were not specifically addressed, as they did not result in clinically relevant symptoms. In the control group, there was one case of surgical vertebral body infection. This patient underwent a purely posterior approach for cement removal, lesion excision, intervertebral implant fusion, and internal fixation and was subsequently discharged. Patients with surgical vertebral body refracture in both groups experienced satisfactory symptom relief following bed rest, brace protection, and standardized antiosteoporotic treatment. Patients with adjacent vertebral body fractures underwent PVP and were discharged after recovery. One patient in the observation group developed hemopneumothorax and another developed pneumothorax; both were treated with closed chest drainage and successfully discharged after recovery (Table 7).

**Table 7:** Complications.

Group	Bone cement leakage	Bone cement displacement	Infection	Recollapse	Adjacent vertebral fractures	Hemothorax or pneumothorax
CG	12	2	1	2	4	0
OG	14	1	0	3	3	2
*χ*2	8.677					
*P*	< 0.001					

## 4. Discussion

Costal pain secondary to OVCF refers mainly to bilateral or unilateral pain bands in the chest and abdomen caused by vertebral fractures [11–13]. Associated conditions that cause similar symptoms, such as rib fractures, shingles, and thoracic surgery, need to be ruled out. The pain can be stabbing, dull, or burning and tends to worsen with coughing or deep breathing, and its incidence can be as high as 30% [10, 13, 14]. Some patients with OVCFs even present with costal pain as their main clinical manifestation [12, 13].

The main treatment modalities for costal pain secondary to OVCFs include conservative approaches such as oral nonsteroidal anti‐inflammatory and analgesic medications [11, 12, 15, 16]. Some scholars believe that the core pathological change in costal pain secondary to OVCFs is the vertebral fracture itself [13, 14]. After vertebral fracture, the intervertebral foramen area is reduced, and the corresponding intercostal nerves are stimulated, possibly leading to pain and numbness in the innervated area [13]. Furthermore, the increased width of the vertebral body after fracture might stimulate the sympathetic ganglia around the vertebrae, leading to thoracic pain [14]. Finally, the loss of anterior spinal column stability after vertebral body fracture and the abnormal movement of the small intervertebral joints are also potential risk factors for costal pain. Based on these theories, some scholars have suggested that costal pain can be relieved by stabilizing the fractured vertebra [7, 8, 15, 16]. Unfortunately, as many as 25% of patients experience significant residual costal pain after simple PVP surgery [10]. The probable reason for this residual costal pain is that PVP alone cannot relieve the soft tissue compression on the nerves in the intervertebral foramen [14, 21, 22]. Prolonged compression and chemical stimulation of the nerves by local inflammatory factors might also contribute to residual costal pain after PVP. Therefore, the efficacy of PVP alone in patients with OVCFs and costal pain remains questionable.

INBs are indicated for multiple chronic and acute pain conditions, including rib fractures, shingles, postthoracotomy pain syndrome, and intercostal neuralgia [17–19]. INB is easy to perform and has few complications, particularly when done under C‐arm navigation during PVP surgery. This study was the first to assess the utility of simultaneous PVP and INB for treating OVCFs with costal pain. Although the operation time and number of C‐arm fluoroscopy uses increased when performing INB during PVP, patients’ costal pain was satisfactorily relieved, and their quality of life significantly improved. Furthermore, adding INB did not significantly increase pleural or lung injury complications, making it a safe and effective treatment option.

Residual postoperative costal pain occurred in 34.8% of patients who underwent PVP alone, which was higher than the previously reported incidence. This difference might be attributed to the larger sample size included in our study. We found that disease duration was an independent risk factor for residual costal pain. This is likely because vertebral fractures, loss of vertebral height, and the ligaments fixing the intercostal nerves inside and outside the intervertebral foramen are important contributors to nerve compression [22]. Moreover, cortical bone fractures and local hematomas can also cause persistent compression of the nerves. Autoimmune reactions and inflammatory factors inevitably lead to further aggravation of nerve damage. As multiple pathogenic factors accumulate with disease prolongation, irreversible nerve damage may occur. Under this premise, it is difficult to immediately relieve intercostal neuralgia through PVP alone. Therefore, we recommend concomitant INB for patients whose disease duration exceeds 15.5 days. PVP with INB, as performed in the observation group of this study, resulted in satisfactory relief of thoracic back and costal pain. However, this was associated with increased operation time and radiation exposure. Given that INB is known to increase the risk of injury to the pleura and adjacent blood vessels, we suggest performing PVP alone for patients with a disease duration of up to 15.5 days.

Some limitations of our study should be noted. This was a retrospective study. Possibly relevant factors—including BMD, body mass index, and the production and release of inflammatory mediators such as interleukins and tumor necrosis factor by injured vertebrae—were not included in our analysis. These inflammatory mediators might stimulate the corresponding nerve roots, resulting in costal pain, an aspect not explored in our current research. We expect that prospective studies with larger samples will validate or refine the findings of this study.

## 5. Conclusions

Costal pain combined with an OVCF in the thoracic spine can be effectively treated by combined PVP and INB. However, the inclusion of INB inevitably increases operation time and radiation exposure risk. PVP alone can provide satisfactory spontaneous relief of costal pain in patients with a disease duration of 15.5 days or fewer; otherwise, concomitant INB is recommended.

## Ethics Statement

This study was approved by the ethics committee of our hospital (KY2025286).

## Consent

The authors have nothing to report.

## Conflicts of Interest

The authors declare no conflicts of interest.

## Author Contributions

S.Z. developed the concept, supervised the project, and conducted data analysis. Z.M. and X.P. conducted the majority of the study, analyzed data, and prepared the manuscript. Z.M. and S.X. conducted sample collection and performed statistical analysis. Q.W. provided critical suggestions and instructions for the project and helped compose the manuscript.

Z.M. and X.P. contributed equally to this study.

## Funding

This work was jointly funded by Sichuan Science and Technology Program (No. 2022‐YFS0628), Research Startup Fund Sponsored Project of Southwest Medical University Hospital (No. 25034), and Natural Science Foundation of Sichuan (No. 2023NSFSC0333).

## Data Availability

The data that support the findings of this study are available from the corresponding author upon request.

## References

[1] Kanis J. A. , McCloskey E. V. , Johansson H. , Oden A. , Melton L. J.3rd, and Khaltaev N. , A Reference Standard for the Description of Osteoporosis, Bone. (March 2008) 42, no. 3, 467–475, 10.1016/j.bone.2007.11.001, 2-s2.0-39149120827. 18180210

[2] Wang Y. , Tao Y. , Hyman M. E. , Li J. , and Chen Y. , Osteoporosis in China, Osteoporosis International. (October 2009) 20, no. 10, 1651–1662, 10.1007/s00198-009-0925-y, 2-s2.0-69949144744. 19415374

[3] Johnell O. and Kanis J. A. , An Estimate of the Worldwide Prevalence and Disability Associated With Osteoporotic Fractures, Osteoporosis International. (December 2006) 17, no. 12, 1726–1733, 10.1007/s00198-006-0172-4, 2-s2.0-33750209491. 16983459

[4] Edidin A. A. , Ong K. L. , Lau E. , and Kurtz S. M. , Morbidity and Mortality After Vertebral Fractures: Comparison of Vertebral Augmentation and Nonoperative Management in the Medicare Population, Spine. (August 2015) 40, no. 15, 1228–1241, 10.1097/BRS.0000000000000092, 2-s2.0-84894029055. 26020845

[5] Zhang H. , Xu C. , Zhang T. , Gao Z. , and Zhang T. , Does Percutaneous Vertebroplasty or Balloon Kyphoplasty for Osteoporotic Vertebral Compression Fractures Increase the Incidence of New Vertebral Fractures? A Metaanalysis, Pain Physics. (2017) 20, no. 1, E13–E28, 10.36076/ppj.2017.1.e13. 28072794

[6] Taylor R. S. , Fritzell P. , and Taylor R. J. , Balloon Kyphoplasty in the Management of Vertebral Compression Fractures: An Updated Systematic Review and Meta-Analysis, European Spine Journal. (2007) 16, no. 8, 1085–1100, 10.1007/S00586-007-0308-Z, 2-s2.0-34248175352. 17277923 PMC2200787

[7] Rad A. and Kallmes D. , Pain Relief Following Vertebroplasty in Patients With and Without Localizing Tenderness on Palpation, American Journal of Neuroradiology. (2008) 29, no. 9, 1622–1626, 10.3174/ajnr.A1186, 2-s2.0-54249111071. 18583403 PMC8118766

[8] Yang Y. M. , Ren Z. W. , Ma W. , and Jha R. K. , Kyphoplasty for the Treatment of Pain Distant to Osteoporotic Thoracolumbar Compressive Fractures, Cell Biochemistry and Biophysics. (2014) 68, no. 3, 523–527, 10.1007/s12013-013-9732-3, 2-s2.0-84896044868. 23959301

[9] Doo T. , Shin D. , Kim H. , Kim H. J. , Chung J. H. , and Lee J. O. , Clinical Relevance of Pain Patterns in Osteoporotic Vertebral Compression Fractures, Journal of Korean Medical Science. (2008) 23, no. 6, 1005–1010, 10.3346/JKMS.2008.23.6.1005, 2-s2.0-60549085839. 19119444 PMC2610635

[10] Gibson J. , Pilgram T. , and Gilula L. , Response of Non-Midline Pain to Percutaneous Vertebroplasty, American Journal of Roentgenology. (2006) 187, no. 4, 869–872, 10.2214/ajr.05.0084, 2-s2.0-33749040220. 16985127

[11] Lin F. , Zhang Y. , Song X. et al., Percutaneous Kyphoplasty to Relieve the Rib Region Pain in Osteoporotic Thoracic Vertebral Fracture Patients Without Local Pain of Fractured Vertebra, Pain Physician. (January 2023) 26, no. 1, 53–59, 10.36076/ppj.2023.26.53. 36791294

[12] Niu J. , Song D. , Gan M. et al., Percutaneous Kyphoplasty for the Treatment of Distal Lumbosacral Pain Caused by Osteoporotic Thoracolumbar Vertebral Fracture, Acta Radiologica. (November 2018) 59, no. 11, 1351–1357, 10.1177/0284185118761204, 2-s2.0-85044252999. 29482346

[13] Chen R. , Zhang P. , Li K. , Liu Q. , and Li G. , Risk Factors of Costal Pain of Thoracic Osteoporotic Vertebral Compression Fractures: Multicenter Retrospective Analysis, Scientific Reports. (March 2025) 15, no. 1, 10.1038/s41598-025-88920-6. PMC11953379 40155612

[14] Xin J. , Liu X. , Jing X. et al., Multifactor Analysis of Costal Pain in Osteoporotic Fracture of Thoracic Vertebra, Pain Physician. (2021) 24, no. 6, E795–E802. 34554699

[15] Tang X. S. , Tan M. S. , Yi P. , Yang F. , Zhao H. , and Yu X. , [Analysis of Clinical Outcome of Kyphoplasty on Costal Pain Related to thoracic Osteoporotic Compression Fractures], Zhong Guo Gu Shang. (September 2017) 30, no. 9, 823–827, 10.3969/j.ISSN.1003-0034.2017.09.008, 2-s2.0-85049263484. 29455483

[16] Choi H. J. , Yang H. J. , Lee S. H. , and Park S. B. , The Effect of Vertebroplasty on Costal Pain Related to Osteoporotic Thoracic Compression Fractures in Elderly Patients, Korean J Spine. (June 2012) 9, no. 2, 98–101, 10.14245/kjs.2012.9.2.98. 25983796 PMC4432368

[17] Williams E. H. , Williams C. G. , Rosson G. D. , Heitmiller R. F. , and Dellon A. L. , Neurectomy for Treatment of Intercostal Neuralgia, Ann Thorac Surg. (2008) 85, no. 5, 1766–1770, 10.1016/j.athoracsur.2007.11.058, 2-s2.0-42949168187. 18442581

[18] Manchikanti L. , Kaye A. D. , Falco F. J. E. , and Hirsch J. A. , Essentials of Interventional Techniques in Managing Chronic Pain, 2018, Springer International Publishing.

[19] Koehler R. P. and Keenan R. J. , Management of Postthoracotomy Pain: Acute and Chronic, Thoracic Surgery Clinics. (2006) 16, no. 3, 287–297, 10.1016/j.thorsurg.2006.05.006, 2-s2.0-33747681552. 17004557

[20] Steyaert A. and Lavand’homme P. , Prevention and Treatment of Chronic Postsurgical Pain: A Narrative Review, Drugs. (March 2018) 78, no. 3, 339–354, 10.1007/s40265-018-0866-x, 2-s2.0-85041125230. 29380289

[21] Court C. , Vialle R. , Lepeintre J. F. , and Tadié M. , The Thoracoabdominal Intercostal Nerves: An Anatomical Study for Their Use in Neurotization, Surgical and Radiologic Anatomy: SRA. (2005) 27, no. 1, 8–14, 10.1007/s00276-004-0281-8, 2-s2.0-15044362474. 15316761

[22] Kraan G. A. , Hoogland P. V. , and Wuisman P. I. , Extraforaminal Ligament Attachments of the Thoracic Spinal Nerves in Humans, European Spine Journal. (April 2009) 18, no. 4, 490–498, 10.1007/s00586-009-0881-4, 2-s2.0-63849236739. 19165508 PMC2899458

